# Dietary Cadmium Exposure and Risk of Breast, Endometrial, and Ovarian Cancer in the Women’s Health Initiative

**DOI:** 10.1289/ehp.1307054

**Published:** 2014-03-14

**Authors:** Scott V. Adams, Sabah M. Quraishi, Martin M. Shafer, Michael N. Passarelli, Emily P. Freney, Rowan T. Chlebowski, Juhua Luo, Jaymie R. Meliker, Lina Mu, Marian L. Neuhouser, Polly A. Newcomb

**Affiliations:** 1 Fred Hutchinson Cancer Research Center, Program in Cancer Prevention, Public Health Sciences Division, Seattle, Washington, USA; 2 Department of Epidemiology, University of Washington School of Public Health, Seattle, Washington, USA; 3 Environmental Chemistry and Technology; and; 4 Wisconsin State Laboratory of Hygiene, University of Wisconsin-Madison, Madison, Wisconsin, USA; 5 David Geffen School of Medicine, University of California, Los Angeles, Los Angeles, California, USA; 6 Department of Epidemiology and Biostatistics, Indiana University School of Public Health, Bloomington, Indiana, USA; 7 Department of Preventive Medicine, Program in Public Health, Stony Brook University School of Medicine, Stony Brook, New York, USA; 8 Department of Social and Preventive Medicine, School of Public Health and Health Professions, State University of New York at Buffalo, Buffalo, New York, USA

## Abstract

Background: *In vitro* and animal data suggest that cadmium, a heavy metal that contaminates some foods and tobacco plants, is an estrogenic endocrine disruptor. Elevated estrogen exposure is associated with breast, endometrial, and ovarian cancer risk.

Objectives: We examined the association between dietary cadmium intake and risk of these cancers in the large, well-characterized Women’s Health Initiative (WHI).

Methods: A total of 155,069 postmenopausal women, 50–79 years of age, who were enrolled in the WHI clinical trials or observational study, participated in this study. We estimated dietary cadmium consumption by combining baseline food frequency questionnaire responses with U.S. Food and Drug Administration data on food cadmium content. Participants reported incident invasive breast, endometrial, or ovarian cancer, and WHI centrally adjudicated all cases through August 2009. We applied Cox regression to estimate adjusted hazard ratios (HRs) and 95% CIs for each cancer, comparing quintiles of energy-adjusted dietary cadmium intake.

Results: Over an average of 10.5 years, 6,658 invasive breast cancers, 1,198 endometrial cancers, and 735 ovarian cancers were reported. We observed no statistically significant associations between dietary cadmium and risk of any of these cancers after adjustment for potential confounders including total dietary energy intake. Results did not differ in any subgroup of women examined.

Conclusions: We found little evidence that dietary cadmium is a risk factor for breast, endometrial, or ovarian cancers in postmenopausal women. Misclassification in dietary cadmium assessment may have attenuated observed associations.

Citation: Adams SV, Quraishi SM, Shafer MM, Passarelli MN, Freney EP, Chlebowski RT, Luo J, Meliker JR, Mu L, Neuhouser ML, Newcomb PA. 2014. Dietary cadmium exposure and risk of breast, endometrial, and ovarian cancer in the Women’s Health Initiative. Environ Health Perspect 122:594–600; http://dx.doi.org/10.1289/ehp.1307054

## Introduction

Cadmium, a carcinogenic heavy metal, is released into the environment as a result of industrial and agricultural activities (Järup and Åkesson 2009). Tobacco, grains, and some vegetables can take up cadmium from soil and concentrate it above soil levels (Alloway et al. 1990; Hellström et al. 2007; Pappas et al. 2006; Peralta-Videa et al. 2009). Therefore, chronic low-level, nonoccupational exposure to cadmium through tobacco smoke and contaminated foods is common. Cadmium inhaled in cigarette smoke is readily absorbed by lung tissue (Agency for Toxic Substances and Disease Registry 2012). Less than 5% of cadmium ingested in food is absorbed, but low iron stores may increase absorption and may partly explain why women are consistently observed to have higher average urine and blood cadmium concentrations than men (Åkesson et al. 2002; Berglund et al. 1994; Vahter et al. 1996). Thus, cadmium exposure may be especially relevant to women’s health (Vahter et al. 2007).

Multiple mechanisms potentially link cadmium to cancer, including oxidative stress and inflammation (Lag et al. 2010; Liu et al. 2009), interference with DNA repair (Asmuss et al. 2000; Giaginis et al. 2006), and alterations of DNA methylation (Takiguchi et al. 2003). More relevant to hormone-related cancers, perhaps, is evidence that cadmium may act on estrogenic signaling pathways (Liu et al. 2008; Stoica et al. 2000), resulting in proliferation of breast cancer cells *in vitro* (Garcia-Morales et al. 1994), and inducing uterus and mammary gland weight increase in rats (Johnson et al. 2003). Long-term treatment with low concentrations of cadmium can malignantly transform breast cells *in vitro*, although the effect appears to be independent of estrogen receptor-α (Benbrahim-Tallaa et al. 2009).

Epidemiologically, occupational studies support a link between cadmium and lung cancer (Stayner et al. 1992; Thun et al. 1985), but have largely not addressed hormone-driven cancers in women. Three nonoccupational case–control studies have observed consistent associations between urinary cadmium and breast cancer risk (Gallagher et al. 2010; McElroy et al. 2006; Nagata et al. 2013). Prospective studies in Sweden showed an association between estimated dietary cadmium and endometrial cancer (Åkesson et al. 2008) and postmenopausal breast cancer (Julin et al. 2012), but not ovarian cancer (Julin et al. 2011). In contrast, similar studies from the United States (Adams et al. 2012a) and Japan (Sawada et al. 2012) did not observe an association of dietary cadmium with postmenopausal breast cancer risk or risk of any cancer, respectively.

In this report we describe our prospective study of dietary cadmium intake and risk of breast, endometrial, and ovarian cancer in the Women’s Health Initiative (WHI).

## Methods

*Study population*. We selected study participants from the WHI, a large longitudinal study of postmenopausal women, 50–79 years of age, comprising observational study (OS) and randomized clinical trial (CT) arms. Details of the study design and recruitment have been extensively described (Anderson et al. 2003; Hays et al. 2003; Women’s Health Initiative Study Group 1998). WHI recruited participants between 1 October 1993 and 31 December 1998 at 40 clinical centers across the United States. A total of 161,808 women enrolled in the WHI.

All participants provided written informed consent. Human subjects review committees at all participating sites approved WHI study protocols. The analyses presented here were reviewed and approved by the Fred Hutchinson Cancer Research Center Institutional Review Board as an ancillary study to WHI, and complied with all applicable U.S. regulations.

*Exposure and covariate assessment*. All women completed questionnaires at baseline screening and enrollment; these included detailed information on demographic characteristics, dietary habits, reproductive history [use of postmenopausal hormones for ≥ 3 months (estrogen or estrogen plus progesterone; pills or patches)], medical history, lifestyle (tobacco use, alcohol use, dietary supplement use), and physical activity (Anderson et al. 2003). Anthropometric measurements were taken at baseline clinic visits using a standardized protocol, and body mass index (BMI; kilograms per meter squared) was calculated as weight divided by height squared.

To assess usual diet, all participants completed a baseline food frequency questionnaire (FFQ), specifically designed for WHI and previously described in detail (Kristal et al. 1997; Patterson et al. 1999). These FFQs captured usual intakes during the prior 3 months of 122 food and beverage line items, comprising 302 individual food and beverage components.

To estimate dietary cadmium intake, we adapted methodology commonly used for dietary micronutrient estimates (Schakel et al. 1997) and described previously for dietary cadmium (Adams et al. 2012a). We used measurements of the cadmium content of foods determined analytically by the U.S. Food and Drug Administration (FDA) as part of the Total Diet Study (TDS) (Egan et al. 2002, 2007; FDA 2013). Briefly, market baskets of 285 or 290 foods were typically purchased each year (1991–2008) from three locations in each of four regions of the United States. These foods were sent to a central laboratory for preparation according to predetermined recipes, and analysis for content of a number of contaminants including cadmium (Egan et al. 2002). Cadmium was determined with graphite furnace atomic absorption spectroscopy; the limit of detection (LOD) varied among food items and ranged from 0.001 to 0.007 mg/kg (Egan et al. 2002).

The arithmetic mean of cadmium content (milligrams per 100 g prepared weight) of all available samples of each food was assigned as the cadmium content for that food. Thus, we averaged over year and region of collection to estimate each food’s cadmium content. To investigate whether this approach ignored important regional or secular trends in the cadmium content in foods, we examined measurements from the 20 foods with the highest reported cadmium concentrations. Regional variation was < 20% of the overall mean for 13 of the 20 top cadmium-containing foods; maximum variation of < 40% was observed for lettuces. No region with systematically higher or lower cadmium values was identified. Year-to-year variation in the 20 foods with highest mean cadmium concentration reached 50% of the mean cadmium. Qualitatively, however, trends with year of measurement were observed for only three of these 20 foods (peanut butter, decreasing; raisin bran cereal, decreasing; and egg noodles, increasing). Because of limited variation by region and year of measurement, we opted to include all available data from the FDA TDS for each food in order to obtain the best estimate of mean cadmium content of food typically consumed in the United States. We did not use baby foods (*n* = 57) in our calculation of dietary cadmium.

We assigned values of zero to individual cadmium measurements for food items below the LOD. For four foods, all measured cadmium values were below the LOD: tap water, olive/safflower oil, martini/palmarosa oil, and vegetable oil. For 127 foods, one or more cadmium measurements fell below the LOD, resulting in an overall mean cadmium content less than the LOD. These foods were primarily meats, fruits and fruit juices, dairy products, and beverages. Overall mean cadmium concentrations values (i.e., averaged over collection years and regions) below the LOD were retained in analysis.

We matched each of 302 food and beverage components comprising the 120 FFQ line items on the WHI FFQ to one of the foods analyzed by the FDA, based on the food names. To allow inclusion of participants at the Hawaii clinical center, 27 additional component foods specific to the Hawaii FFQ were matched. For component foods for which no obviously similar food was analyzed by the FDA, we relied on food “mapping” created by the FDA for the TDS (FDA 2013). In summary, 154 component foods were direct matches based on the food names; 122 component foods were close but differed in either unspecified details (e.g., “summer squash” and “squash”) or preparation (e.g., “raw onion” and “cooked onion”); 26 component foods were matched using the TDS mapping file (e.g., “lentils” and “white beans”). Therefore, we attributed a cadmium content to every component food.

The FFQ analytic program calculated average annual servings of each FFQ line item, adjusted to sex-specific portion sizes, and estimated nutrient intakes based on the University of Minnesota Nutrition Data System for Research (NDSR, version 2007; http://www.ncc.umn.edu/products/ndsr.html). Nutrient and cadmium calculations were performed by the Nutrition Assessment Shared Resource of the Fred Hutchinson Cancer Research Center (Seattle, WA).

*Urinary cadmium and creatinine*. Spot urine samples were collected at baseline from a subset of WHI participants (Anderson et al. 2003). The cadmium concentration in a subset (*n* = 1,050) of urine samples was measured at the Trace Elements Research Laboratory at the Wisconsin State Laboratory of Hygiene (Madison, WI), using sector field inductively coupled plasma mass spectrometry (SF-ICP MS; Thermo-Finnegan, Element 2) as described (Adams et al. 2011; Cheung et al. 2012). Values below the limit of quantification (3.5 ng/L) were assigned values of 2.5 ng/L. Urine creatinine was measured on a Molecular Devices Spectra Max M5e plate reader using BioAssay Systems QuantiChrom Creatinine Assay Kit configured for 96-well plate assays, following manufacturer’s instructions. A modified Jaffe chemistry was employed to quantify picrate-creatinine spectrophotometrically at 510 nm. Samples were run in duplicate; a median coefficient of variation (CV) of 2.7% was observed. The method limit of quantification was 5 μM (0.06 mg/dL).

*Exclusions and missing data*. For these analyses we excluded women with incomplete or invalid (total energy < 600 or > 5,000 kcal/day) FFQ data (*n* = 4,624) or without follow-up information for cancer diagnosis (*n* = 650). We also excluded women with a previous diagnosis of a cancer of interest (breast, *n* = 5,545; endometrial, *n* = 1,005; and ovarian, *n* = 802) from analysis of that specific cancer. For analyses of endometrial cancer risk, we excluded women with hysterectomy before enrollment (*n* = 32,500). For analyses of ovarian cancer risk, we excluded women reporting bilateral oophorectomy before enrollment (*n* = 28,668). We included women with missing information on a given variable as a separate category for adjustment; 10.4% of participants had missing information on one or more variables. A total of 155,069 women were included in one or more analyses (breast cancer, *n* = 150,889; endometrial cancer, *n* = 91,643; ovarian cancer, *n* = 125,569).

*Follow-up for cancer and censoring*. Participants updated medical history annually (OS) or semiannually (CT) through a mailed self-administered or telephone-administered questionnaire. Breast, endometrial, and ovarian cancers reported by participants were adjudicated by WHI Clinical Coordinating Center (WHI-CCC) staff and physician review of medical records (Anderson et al. 2003; Curb et al. 2003). Separate analyses were conducted for each cancer of interest (breast, endometrial, and ovarian). In each analysis, women were followed until the earliest of incidence of the cancer of interest, death, or final contact. The original WHI study period ended on 31 March 2005; subsequent additional active follow-up continued through 2010. Follow-up for this report ended August 2009. For breast cancer analyses, women were censored at incidence of *in situ* breast cancer (*n* = 1,571) and were therefore not included as outcomes but did contribute time-at-risk before *in situ* diagnosis. For endometrial cancer analyses, women were censored at hysterectomy (*n* = 5,872). Hysterectomy was reported on annual or semiannual updates and adjudicated by WHI-CCC for hormone CT participants; analysis of OS participants relied on self-reported hysterectomy information. No updated information on oophorectomy was collected during the study, to enable censoring in analysis of ovarian cancer risk. Death was ascertained through clinical center follow-up of family reports and routine checks with the National Death Index.

*Statistical analyses*. Multivariable Cox proportional hazards regression was applied to estimate adjusted cancer hazard ratios (HRs) with 95% CIs by quintile of dietary cadmium, adjusted for total energy intake by the residual method (Willett and Stampfer 1986). The mean dietary cadmium intake (10.9 μg/day) was added to calculate “energy-adjusted dietary cadmium.” Trends were examined by assigning to each quintile the ordinal value of that quintile and treating it as a continuous variable; *p*-trend is from a Wald test of this coefficient compared with zero in the fully adjusted model.

We selected confounders, each measured at baseline, based on knowledge of risk factors for breast, endometrial, and ovarian cancer, and sources of cadmium exposure. Multivariable models were stratified for enrollment age in bands (50–54, 55–59, 60–69, 70–79 years), and on WHI component (OS or CT), and adjusted for age (years) (in addition to stratification by age band), race/ethnicity (non-Hispanic white, other), education (high school diploma or less; some college or postsecondary education; college degree or more), BMI (< 25, 25–29.9, ≥ 30.0 kg/m^2^), alcohol consumption (drinks/week: none, < 1, 1–6.9, ≥ 7), combined estrogen plus progesterone hormone therapy (never, past user, current user), unopposed estrogen hormone therapy (never, past user, current user), age at first birth (nulliparous, < 2, 20–29, ≥ 30 years), age at menarche (< 12, 12, 13, > 13 years), age at menopause (≤ 42, 43–47, 48–49, 50–52, ≥ 53 years), physical activity (metabolic equivalent hours per week, quartiles), and cigarette smoking history (never, former, current). Breast cancer analyses were additionally adjusted for mammography in the 2 years before baseline (yes/no). In further analyses, we additionally adjusted for daily medium-sized servings of vegetables (< 1.5, 1.5–2.9, ≥ 3), and daily medium-sized servings of grains (quartiles). To investigate alternatives to vegetable servings and grains, we performed additional analyses adjusted for intake of zinc and iron from diet and supplements (quartiles) along with servings of vegetables and servings of grains; or replaced adjustment for servings of vegetables and grains with computed grams of fiber and carbohydrates consumed daily (quartiles). Finally we examined adjustment for pack-years of smoking, in addition to smoking status.

To improve comparability with previous cohorts that have evaluated dietary cadmium and cancer incidence, we applied the same methods for each outcome of interest to selected subgroups of women: never-users of hormone therapy (at enrollment); women with BMI between 18.5 and 25 kg/m^2^; never-smokers; women without diabetes (at enrollment); women in the lowest quartile of zinc intake, iron intake, or servings of grains; women who consumed < 1.5 servings of vegetables/day; and women in the OS. Tests for the statistical significance of interactions, though, are not reported.

In further analyses, we estimated associations between dietary cadmium and breast cancer cases classified according to estrogen receptor status (ER+ or ER–). For analysis restricted to ER+ breast cancer (*n* = 5,161 cases), women were censored at incidence of ER–, borderline, or unclassified breast cancer; conversely, for analyses specific to ER– breast cancer (*n* = 948 cases), women were censored at incidence of ER+, borderline, or unclassified breast cancer.

For this report we estimated the partial Spearman correlation coefficient between creatinine-normalized urine cadmium concentrations and energy-adjusted dietary cadmium estimates, adjusted for age, for 565 never-smokers.

All analyses were completed in Stata Statistical Software, release 12 (StataCorp LP, College Station, TX, USA).

## Results

Estimated dietary cadmium ranged from 0.02 to 59.4 μg/day (mean, 10.9 μg/day; median, 10.3 μg/day), and was higher among women reporting higher levels of vegetable and grain consumption, consistent with foods documented to be high in cadmium, or higher energy intake (Table 1). On average, the major sources of dietary cadmium were vegetables including potatoes (42% of dietary cadmium); grains including bread, pasta, and rice (29%); seafood (2.2%); fruit (3.8%); and meat, poultry, and dairy (3.8%). Dietary cadmium intake varied with many participant characteristics including age, BMI, race/ethnicity, education, smoking history, physical activity, and alcohol consumption. In comparison, estimated dietary cadmium varied only slightly with reproductive history, use of hormone therapy, and mammography utilization before study enrollment.

**Table 1:** Selected baseline characteristics of participants in one or more analyses (total *n* = 155,069), by quintile of estimated total (not adjusted for energy) dietary cadmium exposure [*n* (%)].

Characteristic	Quintile 1 < 7.10 μg/day	Quintile 2 7.10–9.24 μg/day	Quintile 3 9.24–11.35 μg/day	Quintile 4 11.35–14.21 μg/day	Quintile 5 > 14.21 μg/day
Total	31,013 (100)	31,014 (100)	31,014 (100)	31,014 (100)	31,014 (100)
Age (years)
50–54	3,965 (13)	3,867 (12)	4,074 (13)	4,093 (13)	4,667 (15)
55–59	5,858 (19)	5,976 (19)	5,986 (19)	6,384 (21)	6,518 (21)
60–69	13,645 (44)	14,143 (46)	14,196 (46)	14,061 (45)	13,690 (44)
70–79	7,545 (24)	7,028 (23)	6,758 (22)	6,476 (21)	6,139 (20)
WHI study component
Clinical trial	13,212 (43)	13,311 (43)	13,338 (43)	13,361 (43)	13,433 (43)
Observational study	17,801 (57)	17,703 (57)	17,676 (57)	17,653 (57)	17,581 (57)
Total energy intake (kcal/day)^*a*^
600–1,187	21,082 (68)	9,839 (32)	4,749 (15)	2,239 (7)	858 (3)
1,188–1,539	7,308 (24)	11,988 (39)	10,064 (33)	6,413 (21)	2,994 (10)
1,540–1,969	2,198 (7)	7,281(23)	10,932 (35)	11,219 (36)	7,137 (23)
1,970–5,000	425 (1)	1,906 (6)	5,269 (17)	11,143 (36)	20,025 (65)
BMI (kg/m^2^)^*b*^
< 25	10,896 (35)	11,138 (36)	11,064 (36)	10,902 (35)	10,057 (32)
25–29.9	10,744 (35)	10,848 (35)	10,914 (35)	10,621 (34)	10,296 (33)
≥ 30	9,110 (29)	8,757 (28)	8,796 (28)	9,216 (30)	10,374 (33)
Cigarette smoking history^*b*^
Never	15,843 (51)	15,585 (50)	15,700 (51)	15,401 (50)	15,311 (49)
Former	11,648 (38)	12,734 (41)	13,036 (42)	13,553 (44)	13,703 (44)
Current	3,098 (10)	2,283 (7)	1,891 (6)	1,707 (6)	1,581 (5)
Alcohol consumption (drinks/week)^*b*^
None	10,878 (35)	9,097 (29)	8,262 (27)	8,044 (26)	8,540 (28)
< 1	10,456 (34)	10,362 (33)	10,296 (33)	9,841 (32)	9,775 (32)
1–6.9	6,479 (21)	7,878 (25)	8,399 (27)	8,863 (29)	8,542 (28)
≥ 7	2,931 (9)	3,508 (11)	3,896 (13)	4,110 (13)	3,990 (13)
Non-Hispanic white^*b*^	23,469 (76)	25,851 (83)	26,832 (87)	26,934 (87)	26,129 (84)
Education^*b*^
High school or less	13,072 (42)	10,694 (34)	9,372 (30)	8,569 (28)	7,976 (26)
Some college	8,560 (28)	8,722 (28)	8,584 (28)	8,445 (27)	8,415 (27)
College degree	9,121 (29)	11,379 (37)	12,837 (41)	13,789 (44)	14,392 (46)
Mammography^*b*^^,^^*c*^	24,094 (78)	25,144 (81)	25,534 (82)	25,589 (83)	25,438 (82)
Age at first birth (years)^*b*^
Nulliparous	6,744 (22)	6,179 (20)	5,988 (19)	5,827 (19)	6,113 (20)
< 20	4,908 (16)	4,006 (13)	3,600 (12)	3,515 (11)	3,459 (11)
20–29	16,753 (54)	18,129 (58)	18,610 (60)	18,879 (61)	18,487 (60)
≥ 30	2,003 (6)	2,209 (7)	2,369 (8)	2,352 (8)	2,512 (8)
Age at menopause (years)^*b*^
≤ 42	6,324 (20)	5,565 (18)	5,263 (17)	5,093 (16)	5,123 (17)
43–47	5,832 (19)	5,822 (19)	5,780 (19)	5,790 (19)	5,630 (18)
48–49	2,771 (9)	2,898 (9)	2,983 (10)	2,938 (9)	2,851 (9)
50–52	7,887 (25)	8,363 (27)	8,616 (28)	8,501 (27)	8,715 (28)
> 52	6,089 (20)	6,520 (21)	6,697 (22)	7,061 (23)	6,921 (22)
Age at menarche (years)^*b*^
< 12	6,311 (20)	6,606 (21)	6,678 (22)	6,919 (22)	7,397 (24)
12	7,872 (25)	7,995 (26)	8,191 (26)	8,192 (26)	8,116 (26)
13	8,879 (29)	9,045 (29)	9,041 (29)	8,980 (29)	8,836 (28)
> 13	7,803 (25)	7,253 (23)	6,987 (23)	6,821 (22)	6,549 (21)
Unopposed E use^*b*^^,^^*d*^
Never	20,009 (65)	19,970 (64)	19,809 (64)	20,026 (65)	20,135 (65)
Past	4,164 (13)	4,026 (13)	4,018 (13)	3,735 (12)	3,750 (12)
Current	6,819 (22)	6,991 (23)	7,161 (23)	7,241 (23)	7,106 (23)
E + P use^*b*^^,^^*d*^
Never	23,818 (77)	22,918 (74)	22,552 (73)	22,372 (72)	22,407 (72)
Past	2,434 (8)	2,535 (8)	2,786 (9)	2,745 (9)	2,832 (9)
Current	4,749 (15)	5,553 (18)	5,664 (18)	5,890 (19)	5,764 (19)
Physical activity (MET-hr/week)^*b*^
< 2.25	10,502 (34)	8,069 (26)	6,673 (22)	5,632 (18)	4,432 (14)
2.25–8.32	8,162 (26)	8,234 (27)	7,866 (25)	7,534 (24)	6,422 (21)
8.33–17.74	5,990 (19)	7,153 (23)	7,727 (25)	7,962 (26)	8,145 (26)
≥ 17.75	4,675 (15)	5,991 (19)	7,248 (23)	8,527 (27)	10,865 (35)
Servings vegetables^*e*^
< 1.5	23,319 (75)	16,935 (55)	9,071 (29)	3,149 (10)	612 (2)
1.5–2.9	7,043 (23)	12,685 (41)	18,522 (60)	19,142 (62)	9,120 (29)
≥ 3	651 (2)	1,394 (4)	3,421 (11)	8,723 (28)	21,282 (69)
Servings grains^*e*^
< 2.87	19,850 (64)	9,795 (32)	5,206 (17)	2,704 (9)	1,192 (4)
2.87–4.10	8,005 (26)	11,659 (38)	9,880 (32)	6,448 (21)	2,793 (9)
4.11–5.73	2,587 (8)	7,196 (23)	10,565 (34)	11,239 (36)	7,159 (23)
> 5.73	571 (2)	2,364 (8)	5,363 (17)	10,623 (34)	19,870 (64)
Abbreviations: E, estrogen; E + P, estrogen and progesterone postmenopausal hormone therapy; MET, metabolic equivalent; ^***a***^Participants with < 600 kcal/day or > 5,000 kcal/day were excluded. ^***b***^Numbers and percentages do not sum to total due to missing information. ^***c***^Within 2 years before enrollment. ^***d***^As of enrollment, including pills and patches. ^***e***^Daily medium-sized servings.					

Results adjusted only for total energy, age, and WHI study component did not suggest statistically significant dose–response trends in associations between dietary cadmium and any of the three cancers (Table 2). Further adjustment for smoking, BMI, demographics, physical activity, and reproductive history largely left these results unchanged, as did further adjustment for servings of vegetables and servings of grains (Table 2). Notably, the FFQ-derived total energy intake and servings of grains and vegetables were correlated with dietary cadmium (*R*^2^ of 0.4–0.5 for each in univariate analysis). Addition of zinc and iron intake (milligrams per day) to the model did not substantially change results; nor did substitution of daily total fiber (grams) and carbohydrates (grams) for vegetable and grain servings (data not shown). Not adjusting for total dietary energy intake assessed from the FFQ left the interpretation of results substantially unchanged, although some HRs for individual quintiles of dietary cadmium were significantly > 1 for endometrial and ovarian cancer in some but not all models (see Supplemental Material, Table S1). Adjustment for pack-years of smoking did not materially change results (data not shown).

**Table 2:** Adjusted HRs (95% CIs) for breast, endometrial, and ovarian cancer associated with energy-adjusted dietary cadmium exposure.

Outcome and exposure	*n*	Cases	Model 1 HR (95% CI)	*p*-Trend	Model 2 HR (95% CI)	*p*-Trend	Model 3 HR (95% CI)	*p*-Trend
Breast cancer	150,889	6,658
Quintile dietary cadmium
1	30,171	1,198	Reference		Reference		Reference
2	30,185	1,378	0.96 (0.89, 1.04)		0.93 (0.86, 1.00)		0.92 (0.85, 1.00)
3	30,132	1,338	0.98 (0.91, 1.06)		0.94 (0.87, 1.02)		0.93 (0.86, 1.02)
4	30,202	1,416	1.00 (0.93, 1.08)		0.96 (0.89, 1.04)		0.94 (0.86, 1.03)
5	30,199	1,328	0.96 (0.89, 1.03)	0.63	0.93 (0.86, 1.00)	0.20	0.90 (0.81, 1.00)	0.12
Endometrial cancer	91,643	1,198
Quintile dietary cadmium
1	17,589	193	Reference		Reference		Reference
2	18,257	247	0.92 (0.77, 1.11)		0.89 (0.74, 1.07)		0.89 (0.73, 1.07)
3	18,423	231	0.96 (0.80, 1.15)		0.92 (0.77, 1.11)		0.92 (0.75, 1.12)
4	18,747	238	0.91 (0.76, 1.09)		0.87 (0.72, 1.04)		0.86 (0.69, 1.07)
5	18,627	289	0.89 (0.74, 1.06)	0.20	0.86 (0.72, 1.03)	0.12	0.86 (0.67, 1.11)	0.27
Ovarian cancer	125,569	735
Quintile dietary cadmium
1	25,056	123	Reference		Reference		Reference
2	25,091	153	1.15 (0.90, 1.46)		1.12 (0.88, 1.43)		1.05 (0.81, 1.34)
3	25,077	157	1.03 (0.80, 1.31)		1.00 (0.78, 1.28)		0.88 (0.67, 1.15)
4	25,222	138	1.42 (1.13, 1.78)		1.36 (1.08, 1.72)		1.12 (0.85, 1.48)
5	25,123	164	1.05 (0.82, 1.33)	0.22	1.01 (0.79, 1.29)	0.37	0.75 (0.54, 1.03)	0.22
Model 1: adjusted for total energy intake (residual method), age and study component (observational, clinical trial). Model 2: additional adjustment for body mass index, smoking, ­alcohol consumption, race/ethnicity, education, physical activity, age at first birth, age at menarche, age at menopause, unopposed estrogen use, and estrogen and progesterone use. For breast cancer only: also adjusted for mammography 2 years before baseline. Model 3: additional adjustment for daily vegetable servings and daily grain servings. *p*-Trend: Wald test of ordinal variable for quintile of dietary cadmium.								

Associations among the following subgroups of women were generally consistent with those estimated for the cohort as a whole: women with BMI 18.5–25 kg/m^2^; women without diabetes at enrollment; women consuming < 2.5 medium servings of fruits and vegetables and/or < 2.9 medium servings of grains per day (the lowest quartiles); women with zinc intake < 9.0 mg/day (the lowest quartile) from both diet and supplements; women with iron intake < 10.5 mg/day (the lowest quartile) from both diet and supplements; never-users of hormone therapy before enrollment; never-smokers; women with no history of any cancer before enrollment; and participants in the OS only (data not shown). Associations of ER+ and ER– breast cancer subtypes with dietary cadmium intake were similar to overall results for breast cancer (data not shown).

Mean creatinine-corrected urinary cadmium was 0.49 μg cadmium/g creatinine. The Spearman rank partial correlation coefficient (ρ) between energy-adjusted dietary cadmium and creatinine-corrected urinary cadmium, adjusted for age, was 0.085 (*p* = 0.007 for test of null hypothesis that ρ = 0).

## Discussion

Because of the apparent action of cadmium as an endocrine disruptor, or “metallohormone” (Byrne et al. 2009), we investigated the relation between dietary exposure to this potential environmental carcinogen and three hormone-driven cancers. We did not find evidence for an association of dietary cadmium with any of these cancers. Although HRs for breast and endometrial cancer were different from 1 for some quintiles of dietary cadmium, associations based on linear trends were not apparent or tested to be statistically significant. Thus, overall, we interpret our results to provide little evidence of associations between estimated dietary cadmium and risk of breast, endometrial, or ovarian cancer within this large cohort of postmenopausal women.

However, total energy intake and consumption of vegetables and grains, estimated from the FFQ were correlated with dietary cadmium. Thus, we considered whether adjusting for these variables might attenuate the relationship between dietary cadmium and cancer risk through “overadjustment.” Without these adjustments, the endometrial and ovarian cancer HR estimates for the highest versus lowest quintile of dietary cadmium were > 1 and statistically significant in some models. No statistically significant dose–response trends were observed with increasing dietary cadmium exposure, however. Thus, although HR point estimates changed noticeably, overall, our interpretation of the results is largely unchanged.

We took advantage of the large size of the WHI to investigate whether the association between cadmium and cancer in selected subgroups of women was different than associations estimated from the cohort as a whole. We focused on three areas: whether the association between dietary cadmium and hormone-related cancer risk varied with BMI and hormone therapy (Åkesson et al. 2008; Byrne et al. 2009); varied with dietary components such as fruits and vegetables, grains, zinc, or iron that could modulate uptake of dietary cadmium or mitigate the effects of cadmium (Beyersmann and Hartwig 2008; Klaassen et al. 2009; Tallkvist et al. 2001); or varied with tobacco use, a source of cadmium, that could mask an association with dietary cadmium (Åkesson et al. 2008; McElroy et al. 2007; Richter et al. 2009). Furthermore, we conducted separate analyses restricted to ER+ or ER– breast tumors. We found no evidence supporting an association of cadmium with cancer risk in any subgroup examined.

In contrast to our results, prospective studies of postmenopausal women in the Swedish Mammography Cohort observed positive associations between dietary cadmium and risk of postmenopausal breast cancer and endometrial cancer, but not ovarian cancer (Åkesson et al. 2008; Julin et al. 2011, 2012). Interestingly, the association of dietary cadmium with breast and endometrial cancers reported from the Swedish Mammography Cohort was strengthened by adjustment for intake of vegetables and grains (Åkesson et al. 2008; Julin et al. 2012), opposite to our findings. Another study using a dietary cadmium database and FFQ similar to those used for this report, but in a different U.S. population, also observed no association with breast cancer risk (Adams et al. 2012a); and no statistically significant association between dietary cadmium and risk of all cancer, breast cancer, or endometrial cancer was observed in the Japan Public Health Center-based Prospective Study (Sawada et al. 2012). These studies used a methodology similar to that of the present study to estimate dietary intake of cadmium; the average estimated intake of dietary cadmium was similar between the U.S. and Swedish studies (10–15 μg/day), but substantially higher in Japan (27 μg/day). However, differences in the variation in the cadmium content of foods may partially explain differences in results; dietary cadmium estimates may be more accurate or precise in some populations than in others. Three retrospective case–control studies reported a positive association between cadmium exposure and risk of breast cancer (Gallagher et al. 2010; McElroy et al. 2006; Nagata et al. 2013), in contrast to our results. These studies assessed cadmium exposure through measurement of urinary cadmium, believed to be an objective marker of cadmium absorption over decades (Lauwerys et al. 1994; Nordberg and Kjellström 1979), which may explain the discrepant results in comparison to our study. On the other hand, the results from the retrospective case–control studies may be subject to biases that are not present in our prospective study; for example, treatment for breast cancer may influence urinary cadmium, as has been suggested for lead (McElroy et al. 2008). For each of these case–control studies, cases received some treatment before urine sample collection (Gallagher et al. 2010; McElroy et al. 2006; Nagata et al. 2013).

Cadmium is classified as a carcinogen by the World Health Organization (International Agency for Research on Cancer 1993) primarily on the basis of occupational studies. Nonoccupational exposure to cadmium occurs predominantly through tobacco smoke and food (Järup and Åkesson 2009), and the association between environmental cadmium exposure and various cancers has recently received increasing attention. Prospective epidemiological studies have reported associations between cadmium exposure and higher cancer mortality, including endometrial cancer mortality, but not breast or ovarian cancer mortality (Adams et al. 2012b; Menke et al. 2009; Nawrot et al. 2006).

It is possible that our method of exposure assessment resulted in misclassification that may have biased estimated associations toward the null. As described, our methodology was patterned on nutritional epidemiological studies of micronutrients and cancer risk that use a FFQ. Although the FFQ we used in this study was validated for intake of many micronutrients by comparison to daily food records (Patterson et al. 1999), those results may not extend to cadmium. Even if FFQ responses accurately captured food intake, variation in the cadmium content of food items was another potentially important source of measurement error because the amount of cadmium taken up by plants depends on agricultural conditions and crop varietals (Alloway et al. 1990; Arao and Ae 2003; Cataldo et al. 1981; Peralta-Videa et al. 2009). Last, the FFQ measured usual diet close to baseline, which may not be representative of lifetime exposure.

We compared our estimates of dietary cadmium to urinary cadmium concentrations, corrected for creatinine, in a sample of never-smokers in the WHI, and observed a small but statistically significant correlation (ρ = 0.085). Although urinary cadmium has been used to measure low-level environmental exposure in many epidemiological studies, recent reports have suggested that urinary cadmium may not accurately reflect cadmium accumulation in the kidney resulting from long-term, low-level exposure; therefore, urinary cadmium may reflect primarily recent exposure rather than cumulative exposure (Chaumont et al. 2012, 2013). Thus, the correlation between urinary and dietary cadmium may be lacking because these methods assessed cadmium exposure over different periods of time. Overall, measurement error in assessment of dietary cadmium would be nondifferential in this prospective cohort study and could have introduced substantial bias toward a finding of no association (Freedman et al. 2011; Kipnis et al. 2003).

Finally, although we did not assess occupational exposure to cadmium in the WHI, a previous study of the U.S. adult population suggests that elevated cadmium exposure occurs mainly in automotive and electrical repair, mining, metalworking, and similar jobs working directly with metals (Yassin and Martonik 2004). Because the participants in our study were women > 50 years of age, occupational exposure seems unlikely to have been important.

Despite these limitations, our study has substantial strengths, including its prospective design and the large size of the cohort. Our study included triple the number of cases of breast and endometrial cancer risk and nearly double the number of ovarian cancer cases of the largest prior studies from the Swedish Mammography Cohort (Åkesson et al. 2008; Julin et al. 2011, 2012). Furthermore, WHI data on covariates are highly detailed, including only a small percentage of missing information on variables we considered in our analyses. Follow-up of participants through the established WHI-CCC and vital statistics minimized attrition from the cohort through loss to follow-up. Thus, selection resulting from missing data within the cohort, or differential attrition, is unlikely to have substantially biased our results.

## Conclusions

The results of our study did not support the hypothesis that cadmium contamination of food, measured with an assessment of usual diet during the 3 months before baseline, is a risk factor for postmenopausal breast, endometrial, or ovarian cancer, but misclassification of exposure may have attenuated an association. In future prospective studies, alternative assessments of cadmium exposure, such as urinary cadmium concentration, should be tested in relation to risk of hormonal cancers.

## Appendix: Short List of WHI Investigators

***Program Office (National Heart, Blood, and Lung Institute, Bethesda, Maryland):*** Jacques Rossouw, Shari Ludlam, Dale Burwen, Joan McGowan, Leslie Ford, and Nancy Geller. 

***Clinical Coordinating Center (Fred Hutchinson Cancer Research Center, Seattle, WA):*** Garnet Anderson, Ross Prentice, Andrea LaCroix, and Charles Kooperberg. 

***Investigators and Academic Centers:*** JoAnn E. Manson (Brigham and Women’s Hospital, Harvard Medical School, Boston, MA), Barbara V. Howard (MedStar Health Research Institute/Howard University, Washington, DC), Marcia L. Stefanick (Stanford Prevention Research Center, Stanford, CA), Rebecca Jackson (The Ohio State University, Columbus, OH), Cynthia A. Thomson (University of Arizona, Tucson/Phoenix, AZ), Jean Wactawski-Wende (University at Buffalo, Buffalo, NY), Marian Limacher (University of Florida, Gainesville/Jacksonville, FL), Robert Wallace (University of Iowa, Iowa City/Davenport, IA), Lewis Kuller (University of Pittsburgh, Pittsburgh, PA), and Sally Shumaker (Wake Forest University School of Medicine, Winston-Salem, NC).

## Supplemental Material

(139 KB) PDF
