# Supplementary material for: Dietary Cadmium Exposure and Risk of Breast, Endometrial, and Ovarian Cancer in the Women’s Health Initiative
Source: Environ Health Perspect. 2014 Mar 14;122(6):594–600. doi: 10.1289/ehp.1307054 (PMC4050510; doi:10.1289/ehp.1307054)
Supplement: (139 KB) PDF [file ehp.1307054.s001.pdf]

Environmental Health Perspectives doi:10.1289/ehp.1307054

Supplemental Material

Dietary Cadmium Exposure and Risk of Breast, Endometrial, and Ovarian Cancer in the Women's Health Initiative

Scott V. Adams, Sabah M. Quraishi, Martin M. Shafer, Michael N. Passarelli, Emily P. Freney, Rowan T. Chlebowski, Juhua Luo, Jaymie R. Meliker, Lina Mu, Marian L. Neuhouser, and Polly A. Newcomb

Table S1. Adjusted hazard ratio (HR) and 95% confidence intervals (95% CI) for breast, endometrial, and ovarian cancer associated with dietary cadmium exposure, without adjustment for total energy intake.

Outcome and Exposure	N	Cases	Model 1 HR (95% CI)	P-trend	Model 2 HR (95% CI)	P-trend	Model 3 HR (95% CI)	P-trend
**Breast Cancer**	150,889	6,658						
Quintile dietary cadmium								
1	30,171	1,198	Ref.		Ref.		Ref.	
2	30,185	1,378	1.12 (1.04, 1.21)		1.07 (0.99, 1.16)		1.07 (0.98, 1.16)	
3	30,132	1,338	1.08 (0.99, 1.16)		1.01 (0.94, 1.10)		0.99 (0.90, 1.09)	
4	30,202	1,416	1.13 (1.05, 1.22)		1.06 (0.98, 1.14)		1.03 (0.92, 1.14)	
5	30,199	1,328	1.07 (0.99, 1.16)	0.09	1.00 (0.92, 1.08)	0.72[a]	0.96 (0.85, 1.09)	0.39[a]
**Endometrial Cancer**	91,643	1,198						
Quintile dietary cadmium								
1	17,589	193	Ref.		Ref.		Ref.	
2	18,257	247	1.20 (0.99, 1.45)		1.15 (0.95, 1.39)		1.12 (0.91, 1.38)	
3	18,423	231	1.10 (0.91, 1.33)		1.03 (0.85, 1.25)		1.00 (0.78, 1.27)	
4	18,747	238	1.12 (0.92, 1.35)		1.02 (0.84, 1.24)		1.00 (0.76, 1.30)	
5	18,627	289	1.39 (1.15, 1.66)	0.005	1.25 (1.03, 1.50)	0.12	1.25 (0.92, 1.70)	0.40
**Ovarian Cancer**	125,569	735						
Quintile dietary cadmium								
1	25,056	123	Ref.		Ref.		Ref.	
2	25,091	153	1.20 (0.95, 1.52)		1.17 (0.93, 1.50)		1.10 (0.86, 1.42)	
3	25,077	157	1.22 (0.96, 1.54)		1.17 (0.93, 1.49)		1.04 (0.78, 1.39)	
4	25,222	138	1.07 (0.84, 1.36)		1.02 (0.81, 1.32)		0.85 (0.61, 1.18)	
5	25,123	164	1.29 (1.02, 1.63)	0.15	1.23 (0.99, 1.59)	0.33	0.93 (0.64, 1.34)	0.30

Model 1: adjusted for age and study component (observational, clinical trial). Model 2: additional adjustment for body mass index, smoking, alcohol consumption, race/ethnicity, education, physical activity, age at first birth, age at menarche, age at menopause, unopposed E use, and E + P use. For breast cancer only: also adjusted for mammography 2 years prior to baseline. Model 3: additional adjustment for daily vegetable servings and daily grain servings.

P-trend: Wald test of ordinal variable for dietary cadmium.

[a]Inverse trend.
